# Intradialytic Laughter Yoga therapy for haemodialysis patients: a pre-post intervention feasibility study

**DOI:** 10.1186/s12906-015-0705-5

**Published:** 2015-06-09

**Authors:** Paul N Bennett, Trisha Parsons, Ros Ben-Moshe, Merv Neal, Melissa K Weinberg, Karen Gilbert, Cherene Ockerby, Helen Rawson, Corinne Herbu, Alison M Hutchinson

**Affiliations:** Deakin University, Burwood, VIC Australia; Queen’s University, Kingston, ON Canada; Laughlife Wellbeing Programs, Melbourne, VIC Australia; La Trobe University, Bundoora, VIC Australia; Laughter Yoga Australia, Melbourne, VIC Australia; Moorabbin Dialysis Unit, Monash Health, Moorabbin, VIC Australia; Centre for Nursing Research - Deakin University and Monash Health Partnership, Clayton, VIC Australia; Pulmonary Function Laboratory, Clayton, VIC Australia

**Keywords:** Blood pressure, Laughter therapy, Quality of life, Renal dialysis, Respiratory function tests

## Abstract

**Background:**

Laughter Yoga consists of physical exercise, relaxation techniques and simulated vigorous laughter. It has been associated with physical and psychological benefits for people in diverse clinical and non-clinical settings, but has not yet been tested in a haemodialysis setting. The study had three aims: 1) to examine the feasibility of conducting Laughter Yoga for patients with end stage kidney disease in a dialysis setting; 2) to explore the psychological and physiological impact of Laughter Yoga for these patients; and 3) to estimate the sample size required for future research.

**Methods:**

Pre/post intervention feasibility study. Eighteen participants were recruited into the study and Laughter Yoga therapists provided a four week intradialytic program (30-min intervention three times per week). Primary outcomes were psychological items measured at the first and last Laughter Yoga session, including: quality of life; subjective wellbeing; mood; optimism; control; self-esteem; depression, anxiety and stress. Secondary outcomes were: blood pressure, intradialytic hypotensive episodes and lung function (forced expiratory volume). Dialysis nurses exposed to the intervention completed a Laughter Yoga attitudes and perceptions survey (*n* = 11). Data were analysed using IBM SPSS Statistics v22, including descriptive and inferential statistics, and sample size estimates were calculated using G*Power.

**Results:**

One participant withdrew from the study for medical reasons that were unrelated to the study during the first week (94 % retention rate). There were non-significant increases in happiness, mood, and optimism and a decrease in stress. Episodes of intradialytic hypotension decreased from 19 pre and 19 during Laughter Yoga to 4 post Laughter Yoga. There was no change in lung function or blood pressure. All nurses agreed or strongly agreed that Laughter Yoga had a positive impact on patients’ mood, it was a feasible intervention and they would recommend Laughter Yoga to their patients. Sample size calculations for future research indicated that a minimum of 207 participants would be required to provide sufficient power to detect change in key psychological variables.

**Conclusions:**

This study provides evidence that Laughter Yoga is a safe, low-intensity form of intradialytic physical activity that can be successfully implemented for patients in dialysis settings. Larger studies are required, however, to determine the effect of Laughter Yoga on key psychological variables.

**Trial registration:**

Australian New Zealand Clinical Trials Registry - ACTRN12614001130651. Registered 23 October 2014.

## Background

Laughter can provide a positive respite from the adverse emotional effects associated with illness [1] by improving mood [2–4], reducing depression [4–7], and improving life satisfaction [7] and quality of life [4]. These effects have been shown whether laughter has been spontaneous or simulated [7, 8]. Laughter Yoga (LY), developed in India in 1995, is a combination of simulated laughter with Yoga breathing exercises that is typically conducted in a group setting. It incorporates clapping, arm and leg movement, deep breathing exercises, gentle neck and shoulder stretches as well as facilitated laugh and smile exercises [9, 10]. It can improve life satisfaction, subjective wellbeing, and mood, and reduce anxiety and stress in healthy participants [11].

Despite the many psychological benefits of laughter it is unclear if these benefits translate into a positive intervention for people with end stage kidney disease (ESKD) [12]. Given that Laughter Yoga is both a physical and psychological therapy, takes only 30 to 45 min, and is appropriate for group settings [11], it was chosen over other laughter-based and exercise therapies. Thus, the study had three aims: 1) to examine the feasibility of conducting LY for patients with ESKD in a dialysis setting; 2) to explore the psychological and physiological impact of LY for patients with ESKD receiving haemodialysis treatment; and 3) to estimate the sample size that would be required to adequately power a future study.

## Methods

### Design

Pre- and post-intervention feasibility study.

### Participants and setting

This feasibility study was conducted in one satellite haemodialysis centre in Melbourne, Australia. The convenience sample was from the sampling frame of one morning and one afternoon dialysis shift. Justification for the convenience sample and sample size was based on the feasibility aims of the study. Participants were invited to partake in the study if they met the following inclusion/exclusion criteria: people with ESKD receiving haemodialysis, 18 years of age and over, not pregnant, able to understand spoken English, and receiving haemodialysis treatment for greater than three months. People were excluded if they had been hospitalised in the month prior to the study commencing.

### LY Intervention

The LY intervention was delivered during 11 consecutive haemodialysis treatments over a four week period during November and December 2013. Prior to the intervention the LY therapists met with dialysis patients, dialysis nurses and the researchers to tailor the LY intervention to ensure feasibility and safety. The intervention was delivered by two trained and experienced LY therapists with each session taking approximately 30 to 45 min, conducted within the first two hours of haemodialysis treatment.

The intervention consisted firstly of 10 minutes of breathing and stretching exercises: deep belly breathing; body stretching, arms and legs; gentle neck and shoulder stretches, turning the head to the right and left; and smiling to loosen up face muscles; and a throat/chest/belly laugh exercise. The second ten minute section consisted of facilitated laughter exercises, in conjunction with chanting ho, ho, ho, ha, ha, ha, and (non-dialysis arm) clapping or slapping of the thigh. Laughs were facilitated in this order: greeting laugh; handshaking with therapists, and waving to others; Mexican wave; aloha laugh; triathlon laugh, winding up the dialysis machine to make it go faster laugh; clock watching laugh; run out of the ward laugh; shower laugh; brushing teeth laugh; cup of tea laugh. The final 10 min consisted of Laughter Meditation including body relaxing; smiling and relaxing; giggle; freestyle laughter exercises; breathing and relaxing and ending with application of ‘laughter lotion’.

### Measures

#### Psychological variables

Participants were invited to complete a questionnaire prior to the first LY session (T1) and after the last LY session (T2). The questionnaire included measures of general life satisfaction, subjective wellbeing, mood, optimism, control, self-esteem, depression, stress and anxiety. These measures were chosen because they are theoretically consistent with psychological wellbeing and have recently been explored with a non-clinical sample that undertook a laughter yoga intervention [11]. Furthermore, quality of life tools such as the KDQOL are largely symptom-focused and would not be expected to improve with a LY intervention in a feasibility study.

- General Life Satisfaction (GLS) was measured using a single item, “How satisfied are you with your life as a whole?” Participants responded on a 0–10 scale, anchored by 0 = “not at all satisfied” to 10 = “completely satisfied”.
- Subjective Wellbeing (SWB) was measured using the Personal Wellbeing Index (PWI) [13]. This is a 7-item index to assess satisfaction with the domains of life that contribute to general life satisfaction. The domains of life are standard of living, health, achievements, personal relationships, safety, community, and future security. An optional eighth life domain, spirituality or religion, was included for this study. Participants rated their level of satisfaction with each domain on a 0–10 scale as described for GLS.
- Mood was measured using nine affective descriptors specifically selected to represent the four quadrants of Russell’s circumplex model of affect [14]. Participants responded according to how each of the following described their feelings when they think about their life in general on a 0–10 scale, anchored by 0 = “not at all” to 10 = “extremely”. The affective terms were happy, content, tired, active, miserable, alert, enthusiastic, sad, and distressed. Responses to the items ‘happy’, ‘content’ and ‘alert’ were averaged to compute a total score for Homeostatically Protected Mood (HP Mood) [15], reflecting the type of general positive mood that dominates evaluations of subjective wellbeing.
- Optimism was measured using the three optimism items from the Life Orientation Test-Revised (LOT-R) [16]. Participants rated the extent to which they agreed with each item on a 0–10 scale, anchored by 0 = “do not agree at all” and 10 = “completely agree”.
- Control was measured using five items from Pearlin and Schooler’s Mastery scale [17]. Participants rated the extent to which each statement applied to them in general on a scale from 0 = “not at all” to 10 = “extremely”.
- Self Esteem was measured using the five positively worded items from Rosenberg’s Self-Esteem Scale [18]. Participants rated their level of agreement with each item as for the Optimism scale described above.
- Depression, Stress, and Anxiety (i.e., negative emotional states) were measured using the subscales of the DASS-21 [19]. Participants rated the extent to which each statement applied to them over the past week on a scale from 0 = “not at all” to 10 = “extremely”. Though the first subscale is termed ‘depression’ and will be referred to herein as such, the DASS is not a diagnostic tool and this indicator best represents depressed mood.

#### Lung function

Lung function was measured by the Medical Research Council (MRC) dyspnoea scale, forced vital capacity (FVC), and forced expiratory volume in 1 second (FEV1). FEV1% was calculated based on FVC and FEV1 data. Data were collected at five time points. At both the first and the last LY sessions (T1 and T2), lung function was measured twice, including immediately pre- and post-intervention (i.e., T1 pre LY, T1 post LY, T2 pre LY, and T2 post LY). To explore if there were any longer term effects of LY, lung function was measured again one month after the LY intervention ceased which we termed Time Point 3 (T3). Data were collected for 17 patients at the first LY session (T1), 14 patients on the last LY session (T2), and 12 patients one month post-intervention (T3).

#### Blood pressure

Mean arterial pressure (MAP) was calculated as systolic blood pressure + 2 × diastolic blood pressure divided by 3 [20]. Post haemodialysis mean arterial pressure measures were collated retrospectively for all participants at 11 haemodialysis treatments prior to (pre) and 11 haemodialysis treatments during (post) LY intervention within 15 min following the completion of each haemodialysis treatment. Data from the 11 data points were combined to produce a mean pre- and post-MAP for each participant.

#### Intradialytic hypotension (IDH) episodes

The European Best Practice Guidelines definition of intradialytic hypotension, a decrease in blood pressure associated with symptoms requiring an intervention an additional intervention was used to define IDH episodes [21]. The number of IDH episodes recorded for participants in the four weeks prior to the LY intervention (pre), four weeks of the LY intervention (during), and four weeks after the LY intervention (post) was compiled retrospectively.

#### Nursing staff perceptions of LY

Nursing staff in the haemodialysis unit were invited to complete an anonymous 12-item web-based questionnaire to explore their perceptions of the intervention. The author-devised questionnaire contained four demographic questions, five quantitative evaluation items and three open-ended items that allowed participants to provide more in-depth feedback and comments about the intervention.

### Data analysis

Quantitative data were analysed using IBM SPSS Statistics version 22 (IBM Corp, Armonk, NY, USA), including descriptive and inferential statistics. Correlations were used to explore relationships between age and FEV1% and BMI and FEV1%. For parametric data, comparison of pre- and post-intervention data with two time points (i.e., same day FEV1%, MAP) were analysed using paired samples t-tests and those with three time points (i.e., T1 – T3 FEV1%) were analysed using repeated measures analysis of variance (ANOVA). For nonparametric data (i.e., psychological variables), the Wilcoxon Signed Rank Test was used to compare pre- and post-intervention responses. Chi square analysis, including an odds ratio and confidence interval, was used to compare pre- and post-intervention IDH data. Sample size calculations were conducted using G-Power version 3.1, using an alpha value of .05 and power of 80 %. Three psychological variables that were considered most amenable to change following an intervention such as LY (i.e., associated with relaxation and positive mood) were depression, anxiety and stress, all measured using the DASS [19]. These variables represent more transient, temporary states, that are more susceptible to change by intervention, compared to other psychological variables such as self-esteem and subjective wellbeing which are inherently stable over time [22, 23].

### Ethical considerations

This feasibility study was approved by both the Monash Health Human Research Ethics Committee B and the Deakin University Human Research Ethics Committee. All aspects of the research were conducted in accordance with the Declaration of Helsinki and its revisions as well as the National Health and Medical Research Council guidelines of Australia.

## Results

Eighteen patients consented to participate in the study, and 17 participants continued with the intervention for the duration of the study (94 % retention). One 56 year old male withdrew from the study during the first week due to a hospital admission that was unrelated to the study. Thus the final sample included 10 males (58.8 %) and 7 females (41.2 %), who ranged in age from 20–89 years (Mean = 68.06, SD = 17.23) and had a mean body mass index of 24.09 (SD = 5.04, Range = 17.4–37.1).

### Psychological variables

Thirteen out of 17 participants completed the T1 questionnaire (seven males and six females), 8 subsequently completed the T2 questionnaire (five males and three females). One additional participant completed the T2 evaluation though he had not completed the T1 questionnaire. The major reason for these low survey response rates was the researcher’s decision not to further burden the participants who already have a significant chronic disease burden. Table 1 presents the mean scores on all measured variables for participants who completed both the T1 and T2 questionnaires.

**Table 1:** Comparisons of scores on all measured variables from T1 to T2 (n = 8)

	Time 1		Time 2		
Variable	Mean	SD	Mean	SD	Wilcoxon Z test
GLS	71.25	21.67	75.00	17.73	*Z* = −.816, *p > 0.05*
SWB	77.68	12.00	78.57	8.27	*Z* = −.341, *p > 0.05*
HPMood	66.88	24.57	69.58	20.66	*Z* = −.954, *p > 0.05*
Optimism	59.79	27.41	62.50	21.21	*Z* = −.170, *p > 0.05*
Control	40.29	18.67	44.29	20.05	*Z* = −.944, *p > 0.05*
Self Esteem	75.00	17.79	74.75	12.87	*Z* = −.070, *p > 0.05*
Depressed mood	31.79	20.46	34.11	17.60	*Z* = −.284*, p > 0.05*
Anxiety	27.14	15.80	33.04	13.86	*Z* = −1.185, *p > 0.05*
Stress	35.36	21.35	31.07	20.51	*Z* = −.211, *p > 0.05*

Subtle increases were observed for GLS, HPMood, Optimism, and Control, and subtle decreases were also reported for Stress. Both Anxiety and Depression were slightly increased at T2 compared to T1. There was a very minor increase in SWB from T1 to T2. None of these differences were statistically significant.

### Lung function

There was no change in MRC dyspnoea scale scores with identical scores obtained for all patients at T1, T2, and T3. Individual scores ranged from 1 to 4, and the mean score on all occasions was 1.80 (SD = 1.03). The pattern of scores pre and post LY at T1 and T2 suggested that individuals responded differently to the intervention. For example, one person had a FEV1% that increased by 18 points (Pre = 50, Post = 68) between the pre and post intervention data collection at T1, while another participant had a decrease of 18 points (Pre = 95, Post = 77). At T1 (*n* = 17), eight participants recorded a higher FEV1% after LY, eight participants recorded a lower FEV1%, and one participant remained the same pre and post LY. The mean change in FEV1% at T1 was −0.06. At T2 (*n* = 14), six participants recorded a higher FEV1% after LY, seven participants recorded a lower FEV1%, and one participant remained the same. The mean change in FEV1% at T2 was −2.14. There was no correlation between age and lung function or BMI and lung function at any of the five time points. A mean score was calculated for each of the five data collection points (Table 2). There was no immediate effect of the LY on lung function, based on a comparison of pre and post LY mean scores at T1 (i.e., same day FEV1%).

**Table 2:** FEV1% scores across the five data points

Time	*n*	Mean	SD	Minimum	Maximum	t	df	*p*
T1 pre LY	17	63.35	18.03	40	99	0.03	16	0.98
T1 post LY	17	63.29	17.11	29	93			
T2 pre LY	14	64.21	16.87	46	102	0.91	13	0.38
T2 post LY	14	62.07	16.16	43	95			
T3	12	55.17	14.22	26	73			

### Mean arterial pressure (MAP)

Post haemodialysis MAP data was recorded for all participants (*n* = 17) at 11 haemodialysis treatments prior to (pre) and 11 haemodialysis treatments during (post) the LY intervention. Data from the 11 data points were combined to produce a mean pre and post MAP for each participant. Results were mixed with ten patients exhibiting a decrease in their mean MAP between pre and post phases and seven exhibiting an increase. Pre (M = 87.85, SD = 11.72, Range = 72.76 – 115.67) and post (M = 86.25, SD = 8.90, Range = 73.42 – 106.27) MAP data were compared but did not differ significantly, t (16) = 1.14, p = 0.27, η^2^ = 0.07. The mean decrease in MAP was 1.60 with a 95 % confidence interval ranging from −1.38 to 4.58.

### Intradialytic hypotensive (IDH) episodes

There were 19 IDH episodes recorded in the month preceding the LY, 19 IDH episodes recorded during, and four recorded post LY intervention. Based on these data, the odds of IDH decreased by 80 % (OR = 0.20, CI = 0.07 – .61) which was statistically significant (χ^2^_1_ = 9.76, *p* = .002). The reasons for this difference are unclear but it is unlikely that a change of this magnitude would be related to the intervention alone.

### Nursing staff perceptions of LY

Eleven Registered Nurses from the dialysis clinic completed the online questionnaire (61 % response rate) (Table 3). Only one participant had any prior experience with LY and all participants had worked a shift in the unit while the LY study was running. The majority of respondents (82 %) either agreed or strongly agreed that LY had a positive impact on their patients’ mood and that they would recommend LY to future patients. Overall, 9 participants (82 %) were Satisfied or Very satisfied with the LY intervention during dialysis; the remaining two participants selected Neutral.

**Table 3:** Nursing staff responses regarding LY in their haemodialysis unit

Item	Strongly agree	Agree	Neutral	Disagree	Strongly disagree
Laughter Yoga had a positive impact on the mood of patients	5 (45 %)	4 (36 %)	2 (18 %)	0	0
I would recommend Laughter Yoga to my patients	5 (45 %)	4 (36 %)	2 (18 %)	0	0
I would like to have more information about Laughter Yoga	1 (9 %)	5 (45 %)	5 (45 %)	0	0
I had concerns regarding the safety of Laughter Yoga during dialysis^a^	0	1 (9 %)	3 (27 %)	4 (36 %)	2 (18 %)

^a^One participant selected ‘Not applicable’ for this item

### Sample size calculation for future study

Sample sizes were calculated based on the three key psychological variables considered most amenable to change, as follows: Anxiety, *n* = 28; Stress, *n* = 74; and Depression, *n* = 207.

## Discussion

This study demonstrated the feasibility of conducting LY for patients with ESKD in dialysis settings. Despite participant attrition in some components of the evaluation (e.g., lung function, participant survey), the intervention itself recorded a 94 % retention rate over four weeks which indicated that LY was an acceptable intervention for patients. The only withdrawal from the study was a patient who was admitted to hospital for reasons unrelated to the intervention.

Participants reported slight increases in happiness, mood and optimism and had subtle decreases in stress, consistent with LY research in the other populations [24]. For example, feelings of stress were substantially decreased for diabetic patients after participating in LY [24] and feelings of liveliness, activation, cheerfulness, and friendliness increased for patients awaiting organ transplantation [25]. Similarly, our study had a positive impact on wellbeing in terms of improved enthusiasm, positive attitude, ability to laugh for no reason, optimism, stress level, and physical and mental relaxation.

Sample size calculations conducted as part of this study identified that a sample as small as 28 participants may be sufficient to detect a significant change in anxiety, but at least 200 participants would be advisable to detect changes in other key variables. Importantly, although there was minimal attrition from the intervention itself, only 76 % of participants completed the pre-intervention survey and 53 % completed the post-intervention survey; 47 % completed both surveys. This should be taken into account when determining sample size.

We found no significant change in the overall mean post dialysis MAP scores associated with the LY intervention. A randomised controlled trial of a LY intervention for healthy IT professionals found that blood pressure and cortisol level were significantly lower in the intervention group compared with a control group [26] which contrasts with our findings. Furthermore, we found no differences in episodes of IDH during the LY when compared to the previous month’s data. The finding that IDH episodes decreased by 80 % in the month following the LY intervention remains unexplained and although postulation can occur we have no reason to believe that this was associated with LY. One consideration that may be explored is that LY involves the potentially increasing blood pressure effect of exercise with the potentially soothing, blood pressure lowering effect of meditation resulting in no change.

Humour is observed frequently in haemodialysis units [27] so it was not surprising that nursing staff in the haemodialysis unit were supportive of the LY. The support and involvement of nursing staff in the haemodialysis unit is pivotal to the success of such an intervention and was clearly evident in this study, with nurses’ spontaneously participating in each LY session. Nurses’ engagement is particularly important in terms of the long term sustainability of an intervention such as LY.

Although there was no improvement in lung function in this study there was also no significant reduction in lung function. This is particularly pertinent given the mixed findings about the impact of laughter therapies on respiratory function of patients on dialysis, many who suffer pulmonary compromise [28]. One study of patients with severe COPD indicated that humour-induced smiling is beneficial but laughter may be detrimental to patients [29]. Our study did not demonstrate a negative impact on respiratory function.

For patients with ESKD a strong sense of humour has been shown to assist in coping with stressful life events, increasing the likelihood of survival into old age [25]. Adding LY physical exercise may implicate LY as a sustainable laughter-based exercise intervention. This is important because sustainability of any intradialytic physical activity has been rarely successful in the ESKD group [30]. The addition of the meditational relaxation component of LY may also be responsible for the subtle increases in happiness, mood and optimism and the subtle decreases in stress. This meditation component may be particularly useful to reduce any increased anxiety related to the dialysis procedure [31].

### Limitations

Although the sample was small, the 17 participants provided valuable data for analysis and insights into the feasibility of such an intervention for patients in one haemodialysis setting. Further studies with larger samples, and the inclusion of a control group, may provide better results in examining the effects of LY for haemodialysis patients in more diverse settings.

## Conclusion

Evidence to support positive health-related effects of LY is still limited, although this study suggests LY may be a beneficial therapeutic intervention for haemodialysis patients. This study has demonstrated that LY is a safe, inexpensive, accessible and low-intensity form of physical activity which has the potential to improve mood and decrease the anxiety of patients with ESKD in haemodialysis settings. The findings suggest the need for further research in this field with larger samples and controlled designs that may provide a greater understanding of the therapeutic benefits of LY.
